# Varus osteotomy as a salvage procedure for young patients with symptomatic patellofemoral arthritis and valgus malalignment at short- to mid-term follow-up: a case series

**DOI:** 10.1007/s00402-024-05212-w

**Published:** 2024-02-22

**Authors:** Maximilian Hinz, Maximilian Weyer, Moritz Brunner, Lorenz Fritsch, Alexander Otto, Sebastian Siebenlist, Andrea Achtnich

**Affiliations:** Department of Sports Orthopaedics, Technical University of Munich, Ismaninger Strasse 22, 81675 Munich, Germany

**Keywords:** Patellofemoral joint, Patellofemoral osteoarthritis, Joint preservation, Maltracking

## Abstract

**Purpose:**

The purpose of the study was to report the clinical, functional and radiological outcome following varus osteotomy as a salvage procedure in young to middle-aged patients with patellofemoral arthritis (PFA) and associated valgus malalignment. It was hypothesized that a significant improvement in knee function and reduction in pain would be achieved. Moreover, no conversion to patellofemoral joint arthroplasty could be observed.

**Material and methods:**

Patients (< 50 years of age) that underwent varus osteotomy between 08/2012 and 01/2020 for the treatment of symptomatic PFA and associated valgus malalignment were consecutively included (minimum follow-up: 24 months). Patient-reported outcome measures (PROM; International Knee Documentation Committee subjective knee form [IKDC]), Visual Analog Scale [VAS] for pain, Tegner Activity Scale [TAS], and satisfaction with the postoperative results (1–10-scale, 10 = highest satisfaction) and weight-bearing whole-leg anteroposterior radiographs were conducted pre- and postoperatively. The change in PROM and femorotibial angle (FTA) were tested for statistical significance.

**Results:**

In total, 12 patients (14 knees) were included (66.7% female; mean age: 33.8 ± SD 6.6 years). In ten cases, lateral opening-wedge distal femoral osteotomies (DFO) were performed, of which three cases included a concomitant femoral derotation. Three medial closing-wedge DFO and one medial closing-wedge high tibial osteotomy were performed. At follow-up (55.3 ± 29.3 months), a significant improvement in knee function (IKDC: 56.4 ± 14.4 to 69.1 ± 11.2, *p* = 0.015) and reduction in pain (VAS for pain: 3.5 [interquartile range 2.3–5.8] to 0.5 [0–2.0], *p* = 0.018) were observed. Patients were able to reach their preoperative sporting activity level (TAS: 3.0 [3.0–4.0] to 3.5 [3.0–4.0], *p* = 0.854) and were highly satisfied with the postoperative result (9.0 [6.5–10]). Additionally, a significant correction of valgus malalignment was observed (5.0° ± 2.9° valgus to 0.7° ± 3.2° varus, *p* < 0.001). Regarding complications, two re-osteosyntheses were performed due to loss of correction and delayed union. No conversion to patellofemoral arthroplasty occurred.

**Conclusion:**

In patients with symptomatic PFA and associated valgus malalignment, varus osteotomy as a salvage procedure achieved a significant improvement in knee function and reduction in pain. No conversion to patellofemoral joint arthroplasty occurred at short- to mid-term follow-up.

**Level of evidence:**

Retrospective case series, Level IV.

## Introduction

Lateral patellar facetectomy [7], lateral retinaculum lengthening [30], patellofemoral arthroplasty [1, 8, 15, 16, 32], and joint-preserving osteotomies, such as anteromedialization tibial tubercle osteotomy [2], are widely accepted surgical treatment options for patients with symptomatic patellofemoral arthritis (PFA) [20]. Although the etiology of PFA is still debated, PFA is most often found on the lateral aspect of the patellar facet and the corresponding area of the trochlea, and may develop secondary to osteochondral injuries, trochlear dysplasia and/or lateral patellar instability [13, 34]. Common risk factors for the onset of PFA include increasing age, female gender, greater BMI, and coronal malalignment [5, 10, 24, 37].

As coronal limb malalignment is a modifiable risk factor for the onset of PFA, alignment-correcting osteotomies have been proposed to delay the progression of PFA [5]. Following valgus correction in the case of symptomatic patellofemoral instability, an improvement or no progression of cartilage deterioration has been reported [27], which may imply that isolated valgus-correcting osteotomies may be a viable salvage procedure for the treatment of PFA in patients with valgus malalignment. Therefore, varus-producing osteotomies may be an alternative to arthroplasty, particularly in younger patients.

The purpose of this study was to report on the clinical, functional and radiological outcome of patients undergoing varus osteotomy for the treatment of symptomatic PFA and associated valgus malalignment. It was hypothesized that a valgus-correcting osteotomy would lead to a significant improvement in subjective knee function and reduction in pain at short- to mid-term follow-up. Furthermore, an adequate correction of valgus malalignment and a low complication rate would be observed.

## Material and methods

The presented study was approved by the ethics committee of the Technical University of Munich (reference: 2022-178-S) and conducted according to the Declaration of Helsinki. All patients signed written and informed consent forms. Patients (< 50 years of age) that underwent a varus osteotomy for the treatment of PFA between August 2012 and January 2020 at our institution were eligible to participate minimum 24 months postoperatively. Patients with symptomatic lateral knee osteoarthritis and those who underwent a procedure that aimed to address patellofemoral instability and/or patellofemoral arthroplasty were excluded. Patellofemoral arthroplasty was usually reserved for patients aged > 40 years with end-stage PFA and absence of coronal malalignment [13].

### Radiological parameter measurement and surgical planning

Preoperative imaging included magnetic resonance imaging (MRI) of the knee and weight-bearing whole-leg anteroposterior as well as lateral knee radiographs. In nine patients (11 knees) with suspected torsional deformities, either through physical examination or weight-bearing whole-leg anteroposterior radiographs, lower extremity MRI was performed additionally. In our clinical practice, weight-bearing whole-leg anteroposterior radiographs were repeated following alignment-correcting procedures once full weight-bearing is achieved.

The tibial tuberosity–trochlear groove (TT–TG) distance and degree of trochlear dysplasia according to Dejour [25] were evaluated on preoperative MRI. An increased TT–TG was defined as > 20 mm. Type B and D trochlear dysplasias were classified as high-grade [28]. Patellar height was evaluated on lateral knee radiographs and classified using the Caton–Deschamps index with values ≥ 1.2 indicating a patella alta and values ≤ 0.6 indicating a patella infera [6]. For the pre- and postoperative analysis of coronal limb alignment, the femorotibial angle (FTA), mechanical lateral distal femoral angle (mLDFA), and mechanical medial proximal tibial angle (mMPTA) were analyzed on weight-bearing whole-leg anteroposterior radiographs using the medical software mediCAD^®^ (accuracy: 0.01°; mediCAD Hectec GmbH, Altdorf, Germany) according to the method proposed by Strecker [36]. In patients that underwent lower extremity MRI, lower extremity torsion was evaluated according to the method described by Schneider et al. [35]. Due to different surgeons involved in clinical care, the cut-off for a derotational osteotomy usually varied between > 15° and > 20° of femoral antetorsion [12, 18].

A varus osteotomy was indicated in patients with symptomatic PFA and valgus malalignment ≥ 3°. This threshold may have been slightly lower in patients for which a correction of increased femoral antetorsion was performed concomitantly.

### Surgical technique

The type of varus-producing osteotomy performed was based on the underlying bony deformity, as described by Dror Paley [31], as well as an individual risk factor analysis.

**Fig. 1:**
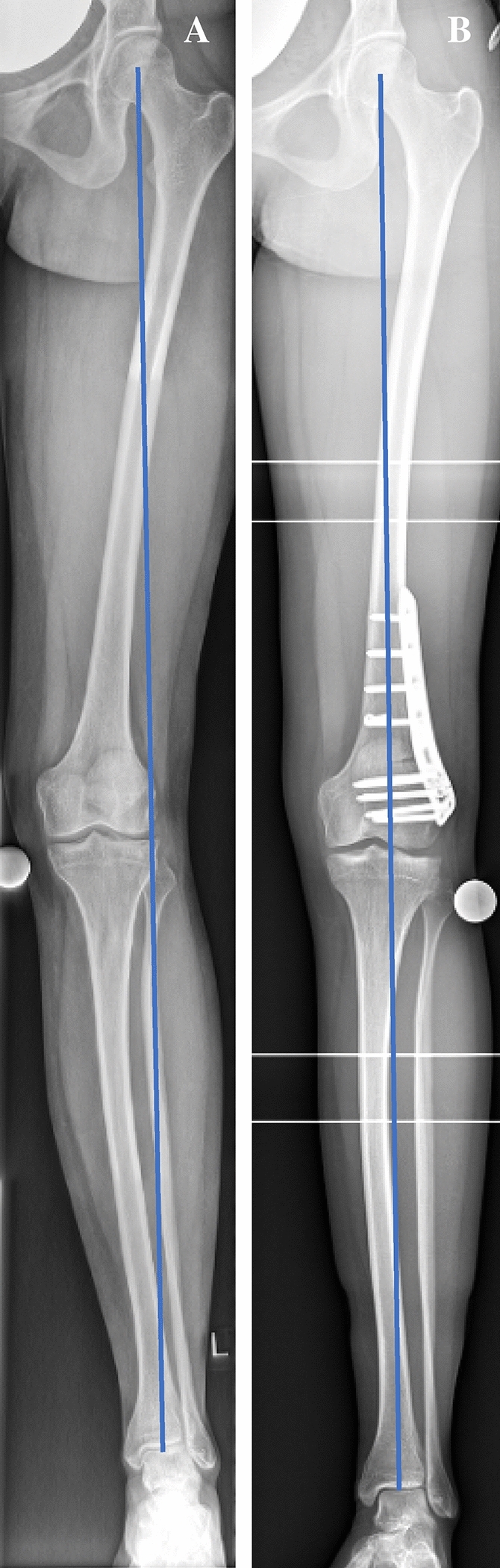
Weight-bearing whole-leg anteroposterior radiographs taken pre- (**A**) and postoperatively (**B**) following a valgus-correcting osteotomy

In patients with femoral deformities, a biplanar supracondylar osteotomy consisting of an osteotomy in the axial and frontal plane of the distal femur was performed via a standardized medial or lateral subvastus approach by experienced sports orthopaedic surgeons. A closing-wedge technique was used in medial osteotomies, whereas an opening-wedge technique was utilized in lateral osteotomies. A medial closing-wedge technique was preferred in patients with a higher risk for delayed union (nicotine abuse, high BMI) [22]. In cases with concomitant torsional deformities, a lateral opening-wedge technique was utilized, as described previously [11, 12]. An internal plate fixator system with locking screws was used (TomoFix^®^ medial/lateral distal femur plate, DePuy Synthes, Umkirch, Germany) for the fixation of the osteotomy, see Fig. 1. Bone grafting of the osteotomy gap was not performed.

In patients with tibial deformities, a biplanar osteotomy in the axial and frontal plane of the proximal tibia was performed via a standardized medial approach. The tibial tubercle was osteotomized distally as to avoid progression of PFA, as recommended in current literature [14, 29]. An internal plate fixator system with locking screws was used (TomoFix^®^ medial high tibia plate, DePuy Synthes, Umkirch, Germany) for the fixation of the osteotomy. The tibial tubercle was fixed using two bicortical screws.

The aim was to achieve neutral alignment in all osteotomies.

### Postoperative rehabilitation

Postoperatively, partial weight bearing (20 kg) was allowed for 6 weeks. After a check-up 6 weeks postoperatively, full weight-bearing was encouraged. Physical therapy started on the first postoperative day with passive flexion with patients undergoing treatments 2–3 times per week. Resumption of daily and sporting activities was recommended after 3 months.

### Outcome assessment

Validated patient-reported outcome measures (PROM; International Knee Documentation Committee subjective knee form (IKDC), Visual Analog Scale [VAS] for pain, Tegner Activity Scale [TAS], subjective satisfaction with the postoperative result [1–10 scale, 10 = highest satisfaction]) were collected pre- and minimum 24 months postoperatively [4]. Return to sporting activities as well as the Kujala score were evaluated at follow-up. Patients were additionally asked whether they would undergo the same procedure again.

Furthermore, demographic factors and patient records with a special focus on revision and conversion to patellofemoral arthroplasty were analyzed.

### Statistical analysis

Data were analyzed using SPSS 26.0 (IBM-SPSS, New York, USA). Categorical variables are presented in counts and percentages. Normal distribution of the collected continuous variables was assessed by the Shapiro–Wilk test and graphically confirmed. Normally distributed continuous variables are shown as mean ± standard deviation. Non-normally distributed continuous variables are shown as median (25–75% interquartile range). For group comparisons of continuous variables, the Wilcoxon test or *t* test was applied. Statistical significance was set at a *p* value of < 0.05.

Due to the rarity of this pathology and the small sample size, all treated subjects were included and a power analysis was not conducted.

## Results

In total, varus osteotomies were performed on 13 patients (15 knees) for the treatment of PFA and associated valgus malalignment between August 2012 and January 2020. Twelve of those patients (66.7% female; 14 knees) were followed-up (92.3%). The remaining patient was excluded from all analyses as no follow-up data were available. At index surgery, patients were 33.8 ± 6.6 years old. Follow-up examinations were conducted 55.3 ± 29.3 months postoperatively. Six patients (50%) reported a history of patellofemoral instability, but patellofemoral pain was the governing symptom in all patients who underwent varus osteotomy.

### Radiological parameters

An increased TT–TG distance was present in one patient (8.3%), who underwent a concomitant medialization of the tibial tubercle. A patella alta was found in three patients (25.0%), whereas no patient had a patella infera. High-grade trochlear dysplasia was observed in five patients (41.7%).

From pre- to postoperatively, mFTA changed significantly (5.0° ± 2.9° valgus to 0.7° ± 3.2° varus, *p* < 0.001).

### Surgical procedures

Ten lateral open-wedge distal femoral osteotomies (DFO) were performed (71.4%). In three of those knees with a mean femoral anteversion of 21.0° ± 4.9°, the osteotomies also aimed to correct femoral antetorsion. Furthermore, three medial closing-wedge DFO (21.4%) and one medial closing-wedge high tibial osteotomy (7.1%) were performed. Concomitant procedures were performed in five knees (35.7%) that underwent DFO, see Table 1. By follow-up, implant removals were performed in 12 knees (85.7%).

**Table 1:** Concomitant procedures (calculation is based on number of knees [*n* = 14])

	No. (%)
Lateral patellar facetectomy	2 (14.3%)
Tripartite patellar fragment excision	2 (14.3%)
Medialization of the tibial tubercle	1 (7.1%)

### Clinical and functional outcome

**Fig. 2:**
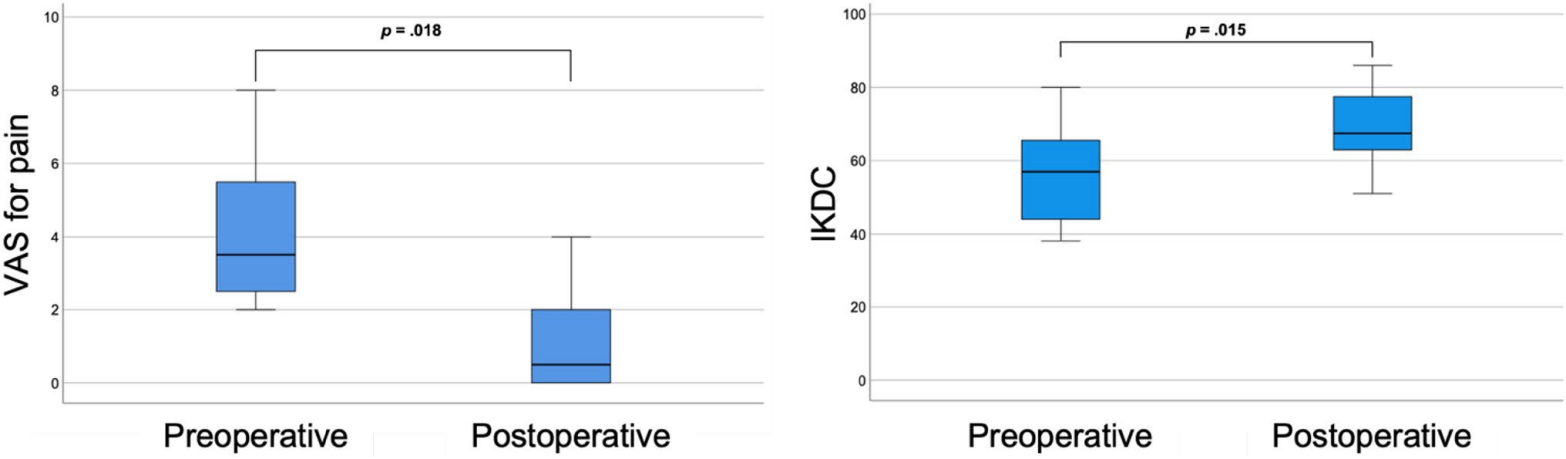
Box-plots comparing pre- vs. postoperative pain levels via VAS for pain (left) and knee function via IKDC (right). Bolded *p* values indicate statistical significance. *VAS* Visual Analog Scale, *IKDC* International Knee Documentation Committee subjective knee form

**Fig. 3:**
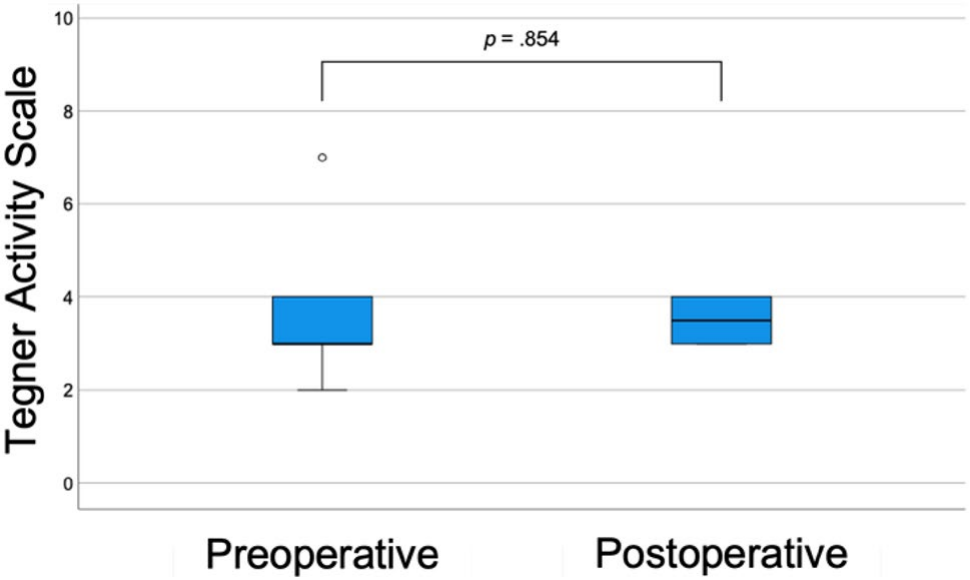
Box-plot comparing pre- vs. postoperative sporting ability via TAS. Bolded *p* value indicates statistical significance. *TAS* Tegner Activity Scale

At follow-up, a significant improvement in knee function (IKDC: 56.4 ± 14.4 to 69.1 ± 11.2, *p* = 0.015) and reduction in pain (VAS for pain: 3.5 [2.3–5.8] to 0.5 [0–2.0], *p* = 0.018) were observed, see Fig. 2. Overall, patellofemoral symptoms were low at follow-up (Kujala score: 87.0 [62.0–92.0] and patients reached the same sporting activity level as preoperatively (TAS: 3.0 [3.0–4.0] to 3.5 [3.0–4.0], *p* = 0.854), see Fig. 3.

Nine patients (75.0% of all included patients) responded to an additional survey on return to sport and work data as well as satisfaction with the postoperative result. Seven patients (77.8%) reported preoperative limitations in their sporting ability. In these patients, sporting ability increased postoperatively in five (55.6%), decreased in one (11.1%) and remained unchanged in three patients (33.3%). Eight patients (88.9%) said that they would undergo the same procedure again. Patients were highly satisfied with the postoperative results (9.0 [6.5–10]). Regarding complications, two re-osteosyntheses were performed 5 and 11 months postoperatively, respectively, due to loss of correction and delayed union. No conversion to patellofemoral arthroplasty or total knee arthroplasty occurred during follow-up.

## Discussion

The most important finding of this study was that patients achieved a significant improvement in knee function and reduction in pain following a valgus-correcting osteotomy for the treatment of PFA and associated valgus malalignment. Additionally, patients were able to return to moderate activity and were highly satisfied with the postoperative result. In total, two complications occurred (14.3%).

Due to the complexity of each case, the treatment of patients with symptomatic, isolated PFA and associated valgus malalignment is challenging, necessitating an individual, risk factor-based approach. This may explain why literature on this topic is limited. Alignment-correcting osteotomies have previously been utilized in treating patients with patellofemoral pain [9, 23], but data for the treatment of PFA is scarce [3]. To our knowledge, only one study previously assessed the outcome following varus osteotomy in patients with symptomatic patellofemoral and lateral tibiofemoral arthritis and associated valgus malalignment [3]. The authors reported a considerable improvement of knee function and significant medialization of the patella. Extrapolation of their data to patients with isolated PFA may, however, be limited due to the presence of lateral tibiofemoral arthritis in their cohort.

Besides varus osteotomies, tibial tubercle osteotomies have been proposed as a treatment option to correct the pathological *Q*-angle—responsible for a laterally translated patella, a shifted force on the lateral patellar facet and, ultimately, increased patellofemoral contact pressure [33]. Following tibial tubercle osteotomy, good results have been reported in patients suffering from PFA [2]. Nevertheless, careful patient selection is paramount as tibial tubercle osteotomies correct a pathological *Q*-angle but not valgus malalignment, which is frequently observed in patients with lateral PFA [5, 24, 37].

Besides an increased *Q*-angle, it has also been shown that increased femoral antetorsion, which is apparent in 30.2% of valgus knees with osteoarthritis [21], may cause an increase in patellofemoral contact pressure. Consequently, in patients with both valgus malalignment and increased femoral anteversion, a varization may be combined with a derotation. Previous biomechanical studies and computer simulation models have reported the effect of derotational osteotomies on coronal alignment. Through some of those studies, concerns were expressed regarding an unintended valgization [17, 19, 26]. In a previous study on patellofemoral instability associated with valgus malalignment and increased femoral antetorsion, however, the authors were able to show that a combined varization and derotation is feasible and accurate using a biplanar supracondylar femoral osteotomy [12]. Consequently, both whole-leg weight-bearing anteroposterior radiographs and lower extremity MRI should be conducted routinely when treating patients with PFA.

This study has several limitations that must be considered. First, its retrospective nature and lack of a control group should be mentioned. The lack of a control group may, however, be due to the unavailability of alternative treatment options. Second, in five knees (35.7%), concomitant procedures were performed which may have confounded the outcome. Third, different types of osteotomies were utilized which was, however, done in an effort to address the underlying bony deformity [31]. Additionally, different cut-off values for increased femoral antetorsion due to multiple surgeons involved and the long inclusion time frame, which may have confounded the outcomes further. Fourth, the IKDC may not have been optimal to assess patients with patellofemoral disorders. As the IKDC is evaluated during our department’s routine follow-up, it was also evaluated during this study to assess the gain in overall knee function rather than the patellofemoral joint, for which the Kujala score was collected only at follow-up. Furthermore, whereas the mean follow-up of 55.3 ± 29.3 months was sufficient to display the short- to mid-term outcome, it may not have been sufficient to evaluate whether patellofemoral joint arthroplasty can be avoided altogether. Nevertheless, the clinical, functional and radiological outcome was favourable and conversion to patellofemoral arthroplasty was not observed within the follow-up period.

## Conclusion

In patients with symptomatic PFA and associated valgus malalignment, varus osteotomy as a salvage procedure achieved a significant improvement in knee function and reduction in pain. No conversion to patellofemoral joint arthroplasty occurred at short-term to mid-term follow-up.
